# Diagnostic Accuracy of the Archimedes Spiral Test for Essential Tremor: A Meta-Analysis

**DOI:** 10.5334/tohm.1151

**Published:** 2026-04-10

**Authors:** Victor Fellipe Bispo Macêdo, Matheus Lang, Jorge Artur Peçanha de Miranda Coelho

**Affiliations:** 1 Universidade Federal de Alagoas, UFAL, Maceió, Alagoas, Brazil; 2 Hospital Santa Casa de Misericórdia de Maceió, Maceió, Alagoas, Brazil

**Keywords:** Essential tremor, Archimedes spiral, diagnostic accuracy, digital biomarkers, artificial intelligence, meta-analysis

## Abstract

**Background:**

The Archimedes spiral test is widely used in clinical neurology to evaluate tremor, yet its diagnostic performance for essential tremor (ET) remains unclear and methodologically inconsistent across studies.

**Objective:**

To determine the diagnostic accuracy of the Archimedes spiral test for distinguishing ET from healthy controls (HC) and to identify methodological and technological factors associated with improved performance.

**Methods:**

A systematic review and meta-analysis were conducted according to PRISMA 2020 guidelines (PROSPERO: CRD420251167793). Eight studies (1,046 participants) were included. Risk of bias was assessed with QUADAS-2. Pooled diagnostic accuracy was estimated using a random-effects model, and subgroup/meta-regression analyses examined the impact of digital acquisition, AI algorithms, EMG/accelerometry, and execution parameters.

**Results:**

The pooled diagnostic accuracy was 0.89 (95% CI: 0.77–0.95; I² = 75.4%). Technology-assisted methods (digital capture or AI-based analysis) demonstrated higher accuracy (0.91; 95% CI: 0.79–0.96) than manual approaches (0.78; 95% CI: 0.70–0.84), although differences did not reach statistical significance. Exploratory analyses suggested that medication withdrawal, absence of arm support, digital recording, and standardized execution protocols may contribute to improved diagnostic performance.

**Conclusions:**

The Archimedes spiral shows moderate-to-high diagnostic accuracy for ET, particularly when combined with digital acquisition and objective analytic methods. Given the small number of available studies and substantial methodological heterogeneity, these findings should be interpreted as exploratory. Standardized digital protocols may enhance reproducibility and support the development of scalable, clinic-ready tremor biomarkers.

## 1. Background

Essential tremor (ET) is among the most common movement disorders worldwide [1], with prevalence increasing substantially with age [2], as highlighted by updated global prevalence estimates from a recent comprehensive review by Louis and McCreary [3]. Its diagnosis remains primarily clinical [4], with absence of a definitive biomarker. Bedside pen-and-paper tasks are frequently used to support diagnostic reasoning, but few have undergone formal validation as diagnostic instruments. Among them, the Archimedes spiral is widely employed due to its simplicity, low cost, and ability to capture tremor amplitude and irregularity [5,6,7,8].

Recent technological advances — including digital acquisition devices, signal-processing techniques, and artificial intelligence — have expanded the potential of spiral analysis to provide objective and reproducible metrics [9,10,11,12,13,14]. However, significant variability exists across studies regarding spiral execution, recording methods, and scoring approaches, limiting conclusions about its true diagnostic accuracy.

To address this gap, we conducted the first systematic review and meta-analysis aimed at determining the diagnostic performance of the Archimedes spiral in distinguishing ET from healthy controls, and at identifying methodological and technological factors associated with improved accuracy.

## 2. Methods

The review was conducted according to the PRISMA 2020 statement (see Supplementary File 1) [15] and was prospectively registered in PROSPERO (CRD420251167793).

### 2.1. Search strategy and studies selection

A comprehensive search was performed in MEDLINE/PubMed, Cochrane Library, EMBASE, SCOPUS, ScienceDirect, ClinicalTrials.gov and ISI Web of Science between June 8 and June 10, 2024. The strategy combined controlled vocabulary and keywords related to “essential tremor”, “spirography”, “Archimedes spiral”, and “spiral” (The full search strategy for each database is provided in Supplementary File 2). Additional studies were identified through manual reference checking and the Research Rabbit discovery platform [16].

All retrieved records were imported into Rayyan® to facilitate duplicate detection and screening [17]. Two reviewers (VM, ML) independently evaluated titles and abstracts. Full texts were retrieved for studies meeting inclusion criteria or when eligibility remained uncertain. Disagreements were resolved through discussion with a third reviewer (JA).

### 2.2. Inclusion and exclusion criteria

Studies were included if they: (1) were original research; (2) enrolled adults (≥18 years) with essential tremor (ET) and a healthy control (HC) group; (3) used the Archimedes spiral as a diagnostic or discriminative tool; and (4) included at least 10 participants. And was excluded if they are: (1) reviews, case reports, conference abstracts, or meta-analyses; (2) studies not published in English or Portuguese; and (3) inability to extract ET-specific results when other tremor syndromes were included. Final eligibility was determined by consensus.

### 2.3. Data extraction and organization

Data extraction was performed by one investigator (VM) using standardized tables and independently verified by a second reviewer (ML). Extracted data included: study characteristics and design, population characteristics, diagnostic criteria for ET, spiral execution parameters (hand used, drawing direction, arm support, medication withdrawal), recording method (paper vs digital), analytic approach (clinical scales, signal processing, artificial intelligence), and diagnostic accuracy metrics (sensitivity, specificity and accuracy). When not explicitly reported, accuracy was determined based on standard diagnostic performance indicators provided by each study.

When essential information was missing, corresponding authors were contacted by email (three attempts at two-week intervals). When no response was obtained, studies were retained if core diagnostic accuracy data were available.

### 2.4. Quality assessment and risk of bias

Risk of bias and applicability concerns were assessed independently by two reviewers using the QUADAS-2 tool [18]. Each domain—patient selection, index test, reference standard, and flow and timing—was categorized as “low”, “high”, or “unclear” risk. Disagreements were resolved by consensus with a third reviewer (JA).

### 2.5. Statistical analysis

Descriptive statistics were obtained using Jamovi (version 2.6.2.0). Categorical variables related to participants and spiral execution and recording parameters were explored using Fisher’s exact test, with corresponding effect sizes (Cramer’s V or odds ratios). Due to small subgroup samples and sparse tables, these analyses were interpreted as exploratory. Statistical significance set at *p* < 0.05.

### 2.6. Meta-analysis

Seven of the eight eligible studies provided sufficient data for quantitative synthesis. A random-effects model was used to estimate pooled diagnostic accuracy. Heterogeneity was assessed using the I² statistic, τ², and Cochran’s Q test. Publication bias was explored through visual inspection of funnel plots; formal statistical testing was not performed due to the small number of studies (n < 10). Analyses were conducted using R software (version 4.2) with the meta package.

Sensitivity analysis was conducted using a leave-one-out approach. Subgroup analyses and weighted meta-regression evaluated the impact of digital acquisition tools, artificial intelligence methods, and additional recording devices (accelerometry, EMG).

To evaluate the impact of technology use on diagnostic accuracy, a simple linear regression weighted by the number of participants was conducted using analysis of variance (ANOVA) to determine the overall significance of the association.

Receiver operating characteristic (ROC) curves were constructed using sensitivity and specificity data when available.

## 3. Results

### 3.1. Study selection and characteristics

**Figure 1:**
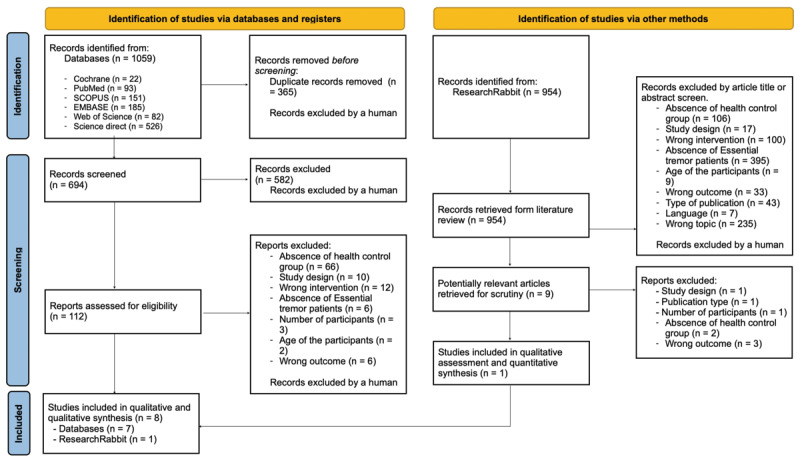
PRISMA flowchart.

A total of 1059 records were identified across six databases, of which seven studies met inclusion criteria, and 954 studies were identified through Research Rabbit platform, which 01 additional study met inclusion criteria (Figure 1). In total, eight studies were included in the qualitative and quantitative synthesis, seven provided sufficient data for meta-analysis.

These studies included 1046 participants, of whom 34.3% belonged to the ET group and females represented 53% of the total sample. Diagnostic criteria for ET were heterogeneous: 62.5% used the 2018 MDS clinical diagnostic criteria [4], 25% used the MDS 1998 criteria [22], and one study did not specify its diagnostic standard. The main characteristics of included studies are summarized in Table 1.

**Table 1:** Summary of included studies.


STUDY (YEAR)	DESIGN	LOCAL	PARTICIPANTS			MEN IN TE GROUP	AGE (SD)		IRB
	
			TOTAL	ET	HC		ET	HC	

**Lorenz et al (2008) [19]**	*Cross-sectional*	Denmark/Germany	624	190	346	122	74,7 (SI)	74,1 (SI)	Yes

**López-de-ipiña et al (2015) [26]**	*Cross-sectional*	Spain	51	24	27	WI	WI	WI	No

**López-de-ipiña et al (2016) [27]**	*Cross-sectional*	Spain	51	24	27	WI	WI	WI	Yes

**López-de-ipiña et al (2018) [23]**	*Feasibility study*	Spain	50	29	21	WI	WI	WI	Yes

**Jordi Solé-Casals et al (2019) [28]**	*Cross-sectional*	Spain	24	12	12	WI	WI	WI	Yes

**Ishii et al (2020) [13]**	*Cross-sectional*	Japan	108	36	36	15	73(12,4)	67,4(10,9)	Yes

**Roth, Brain-Beyamin, Rosenbaum (2021) [20]**	*Cross-sectional*	Israel	38	20	18	10	64,9(15,7)	64,4(10,9)	Yes

**Rajan et al (2023) [21]**	Validação diagnóstica	India	100	25	25	22	52,5(15,2)	42,8(15,4)	Yes


ET – Essential tremor; HC – Health control; SD – standard deviation; IRB – Institutional Review Board; WI – Without information.

### 3.2. Risk of bias

The assessment of bias and applicability concerns using QUADAS-2 revealed an overall low risk, despite the clear absence of data for a complete and thorough evaluation, except for patient selection, for which the risk was mostly classified as unclear.

### 3.3. Execution and evaluation of the Archimedes spiral

The execution and assessment characteristics of the Archimedes spiral test are summarized in Table 2. Considerable methodological variability was observed across studies.

**Table 2:** Archimedes spiral characteristics summary.


STUDY (YEAR)	HAND USED	ATTENUATING	ARM	DRAWING	START	DRAWING	ACQUISITION	OTHER DEVICES	AI USE	EVALUATION FORM	APPLIED SCALE	SENSIBILITY	SPECIFICITY ACCURACY	

		MEDICATION	SUPPORT	DIRECTION	POINT	SHAPE	METHOD							

**Lorenz et al(2008) [19]**	Dominant	–	–	–	Inner	Guided	Paper	No	No	Amplitude + Regularity	BFS	52.60%	75.50%	74.58%

**Lôpez-de- ipina et al (2015) [26]**	Most affected	–	–	–	–	–	Tablet	No	Yes	Linear characteristics + Entropy	–	94.10%	92.15%	92.97%

**Lôpez-de-ipina et al (2016) [27]**	Best performance	–	–	Clockwise	–	Free	Tablet	EMG + Accelerometer	Yes	Linear characteristics + Entropy	FTM	–	–	96.07%

**Lôpez-de-ipina et al (2018) [23]**	Both Hand	–	–	Clockwise	–	–	Tablet	No	Yes	Fractal dimension + Entropy	FTM	–	–	94.11%

**Jordi Solé- t’asalset al (2019) [28]**	Both Hand	–	–	–	–	–	Tablet	No	Yes	DCT + Residual + Radius	WHIGET	100%	96.42%	97.96%

**Ishii et al (2020) [13]**	–	–	–	–	Outer	Guided	Paper	No	Yes	Mean deviation + Percentage of spiral length	TETRAS	44%	79%	70%

**Roth, Brain-Beyamin, Rosenbaum (2021) [20]**	–	–	Yes	Clockwise	–	Guided	Tablet	No	No	Radial deviation + Drawing speed	TETRAS	–	–	–

**Rajan et al (2023) [21]**	Dominant	No	No	Clockwise	Inner	Free	Paper	EMG + Accelerometer	Yes	Mean deviation + Tremor variability	BFSRS	80.90%	76.00%	78.45%


FTM – Fahn-Tolosa-Marin; BFS – Bain-Findley spiral Rating Scale; TETRAS – Tremor Research Group Essential Tremor Rating Assessment Scale; WHIGET – Washington. Heights-Inwood Genetic Study of Essential Tremor; DCT – Discrete Cosine Transform.

### 3.4. Diagnostic accuracy

**Figure 2:**
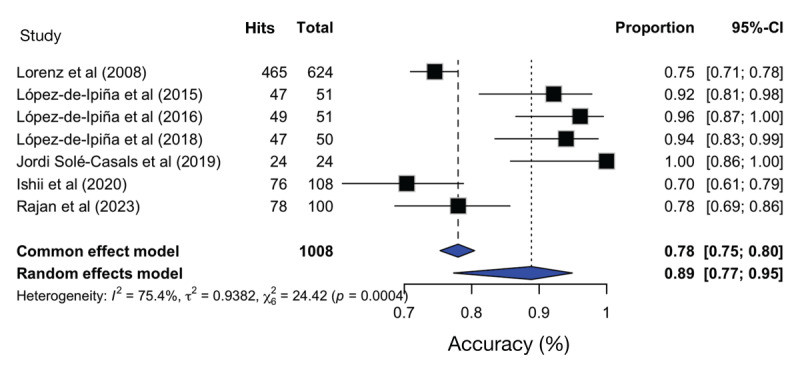
Forest-plot of global ET diagnosis accuracy. *I*^2^ – Heterogeneity test; τ^2^ – Variance between studies; CI – Confidence interval.

Diagnostic accuracy information was available in seven studies, ranging from 70% to 97.96%. The pooled diagnostic accuracy for distinguishing ET from healthy controls was 0.89 (95% CI: 0.77–0.95) across seven studies (Figure 2). Heterogeneity was substantial (I² = 75.4%; τ² = 0.9382; Q test p < 0.001). Five studies provided areas under the ROC curve (AUC), with values ranging from 0.77 to 0.96.

### 3.5. Subgroup analyses

Exploratory subgroup comparisons suggested higher diagnostic accuracy in studies using digital recording, signal-processing approaches, or artificial intelligence (accuracy = 0.91; 95% CI: 0.79–0.96) compared with traditional pen-and-paper methods (accuracy = 0.78; 95% CI: 0.70–0.84). However, differences did not reach statistical significance (p = 0.314).

### 3.6. Meta-regression

Meta-regression did not identify any single methodological variable that significantly explained heterogeneity (p > 0.05 for all predictors). This reflects the limited number of available studies and variability across analytic methods.

### 3.7. Sensitivity and publication bias analyses

**Figure 3:**
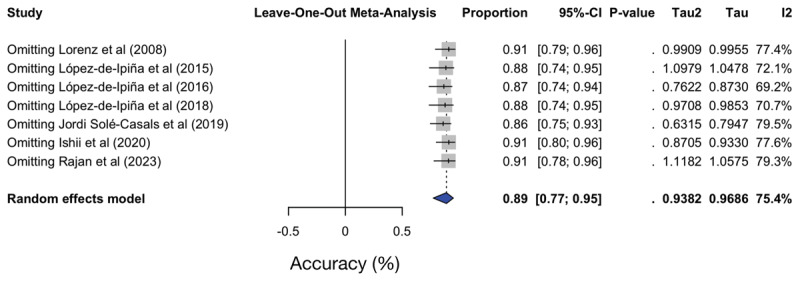
Sensitivity analyses. *I*^2^ – Heterogeneity test; τ^2^ – Variance between studies; CI – Confidence interval.

Leave-one-out sensitivity analysis showed that pooled accuracy estimates remained stable, with a range of 0.86 to 0.91, with no individual study exerting a disproportionate influence on overall results, confirming the robustness of findings (Figure 3). Visual inspection of the funnel plot did not suggest marked publication bias, although the small number of studies (n < 10) limits interpretability.

## 4. Discussion

In this systematic review and meta-analysis, the Archimedes spiral demonstrated moderate-to-high diagnostic accuracy for distinguishing essential tremor (ET) from healthy controls. These findings reinforce long-standing clinical impressions that spiral drawing captures meaningful features of tremor amplitude and irregularity that are visually recognizable at the bedside [4,19]. However, the substantial heterogeneity observed across studies highlights the lack of standardized procedures for spiral execution, recording, and interpretation—an issue repeatedly emphasized in the ET literature [20,21].

Our exploratory subgroup analyses showed a consistent trend toward higher diagnostic accuracy in studies using digital acquisition systems, signal-processing techniques, or artificial intelligence–based analysis. These approaches reduce subjectivity, improve reproducibility, and extract kinematic characteristics not visible to the naked eye [22,23,24]. Although these comparisons did not reach statistical significance—likely because of small sample sizes and methodological variability—the direction of effect aligns with broader developments in digital biomarkers for movement disorders [25,26].

Execution parameters also appeared to influence diagnostic performance. Differences in arm support, drawing direction, or medication withdrawal can modify tremor amplitude and regularity, altering the recorded signal. Similar effects have been described in other standardized motor tasks used in ET research [27,28]. Nonetheless, inconsistent reporting across studies prevented a more detailed assessment of which parameters are most influential. Harmonized protocols that explicitly describe these variables are essential for improving reproducibility and enabling valid comparisons across clinical and research environments. In particular, methodological factors such as hand support and drawing paradigm substantially influence tremor ratings and their reproducibility, as demonstrated by Ondo et al [29].

The clinical implications of these findings are notable. The Archimedes spiral is widely accessible, inexpensive, and familiar to clinicians, making it an attractive candidate for primary care screening, telemedicine evaluation, and longitudinal monitoring of ET. Digital spirals—particularly those acquired using tablets or mobile devices—have the potential to expand this utility further by allowing automated scoring and remote quantification of tremor severity [22,25]. However, broader adoption will require the establishment of standardized execution procedures and validated diagnostic thresholds.

This review has limitations. The number of eligible studies was small, and heterogeneity was substantial. Although we used a random-effects model and performed sensitivity and exploratory analyses, pooled estimates should be interpreted cautiously. Diverse diagnostic criteria, analytic techniques, and recording devices further limit the precision of quantitative synthesis [20,21,22]. Publication bias cannot be excluded, although visual inspection of the funnel plot did not suggest major asymmetry.

Another important factor influencing the diagnostic performance of spiral analysis is tremor severity among included ET cases. Studies enrolling a higher proportion of mild ET, such as population-based cohorts, would be expected to yield lower discriminative accuracy compared with clinic-based samples enriched for moderate-to-severe disease. Unfortunately, most included studies did not systematically report baseline tremor severity, precluding stratified or sensitivity analyses according to disease intensity.

In addition, current consensus diagnostic criteria for ET do not specify a minimum tremor severity threshold, allowing inclusion of very mild cases that may resemble controls. This may further reduce the apparent validity of spiral-based metrics and should be considered when interpreting pooled estimates.

Furthermore, ET manifestations vary across motor tasks, and bedside tests differ in their ability to elicit tremor. Prior work has demonstrated that spiral drawing may be less sensitive than other motor tasks in detecting tremor, as finger-to-nose test which has identified tremor in 88% of ET cases, particularly in milder cases [30]. Consequently, spiral analysis should not be viewed as a standalone diagnostic tool but rather as part of a broader multimodal assessment battery.

Despite these limitations, this review provides the most comprehensive synthesis to date of the diagnostic accuracy of the Archimedes spiral for ET. The results underscore both its promise and the need for consensus-based digital protocols to standardize acquisition and analysis. Future multicenter studies using harmonized digital spirals and rigorous reporting standards will be critical for developing reproducible, scalable, and clinically meaningful spiral-based biomarkers for ET.

## 5. Conclusion

In this systematic review and meta-analysis, the Archimedes spiral demonstrated moderate-to-high diagnostic accuracy for distinguishing essential tremor from healthy controls. Although the performance was influenced by substantial methodological heterogeneity, studies using digital acquisition and objective analytic approaches tended to show improved accuracy. These findings highlight the promising role of the spiral as an accessible, low-cost tool for tremor assessment, particularly when integrated with modern digital technologies. However, the lack of standardized execution protocols and inconsistent reporting remains a major barrier to reproducibility. Future multicenter studies employing harmonized digital spirals, transparent methodology, and validated analytic frameworks are essential to establish robust diagnostic thresholds and advance the role of spiral-based biomarkers in movement disorders.

## Additional Files

The additional files for this article can be found as follows:

10.5334/tohm.1151.s1 Supplementary File 1. PRISMA 2020 Checklist.

10.5334/tohm.1151.s2 Supplementary File 2. Full Search Strategy.

10.5334/tohm.1151.s3 Supplementary File 3. QUADAS-2 Detailed Table.
