# Supplementary material for: Diagnostic Accuracy of the Archimedes Spiral Test for Essential Tremor: A Meta-Analysis
Source: Tremor Other Hyperkinet Mov (N Y). 2026 Apr 10;16:25. doi: 10.5334/tohm.1151 (PMC13068091; doi:10.5334/tohm.1151)
Supplement: Supplementary File 2. — Full Search Strategy. [file tohm-16-1-1151-s2.pdf]

Databases Searched

- MEDLINE/PubMed
- EMBASE
- SCOPUS
- ScienceDirect
- Cochrane Library
- ISI Web of Science
- ClinicalTrials.gov

Search dates: 8–10 June 2024

1. PubMed Search Strategy

("Essential Tremor”)
AND
("Spiral" OR "Archimedes spiral" OR "spirography")

2. EMBASE

('essential tremor’)
AND
('archimedes spiral’ OR ‘spiral' OR 'spirography')

3. SCOPUS

("essential tremor")
AND
("archimedes spiral" OR "spiral" OR "spirography")

4. Web of Science (ISI)

(“essential tremor")
AND
("archimedes spiral" OR "spiral" OR "spirography")

5. Cochrane Library

(“essential tremor")
AND
("archimedes spiral" OR "spiral" OR "spirography")

6. ScienceDirect

(“essential tremor")
AND
("archimedes spiral" OR "spiral" OR "spirography")

7. ClinicalTrials.gov

Advanced Search fields:

- Condition/Disease: "Essential Tremor"
- Other Terms: "spiral" OR "Archimedes spiral" OR "spirography"
- Study Type: All
- Status: All
