# Supplementary material for: Diagnostic Accuracy of the Archimedes Spiral Test for Essential Tremor: A Meta-Analysis
Source: Tremor Other Hyperkinet Mov (N Y). 2026 Apr 10;16:25. doi: 10.5334/tohm.1151 (PMC13068091; doi:10.5334/tohm.1151)

Study	RISK OF BIAS				APPLICABILITY CONCERNS		
	PATIENT SELECTION	INDEX TEST	REFERENCE STANDARD	FLOW AND TIMING	PATIENT SELECTION	INDEX TEST	REFERENCE STANDARD
Lorenz et al (2008)	☹	☺	☺	?	?	?	?
López-de-ipiña et al (2015)	?	☺	☺	☺	?	?	?
López-de-Ipiña (2016)	?	?	?	☹	☺	☺	☺
López-de-Ipiña (2018)	?	?	?	?	☺	☺	☺
Jordi Solé-Casals et al (2019)	?	☺	?	☺	☺	☺	☺
Ishii et al (2020)	☺	☺	☺	☺	☺	☺	☺
Roth, Brain-Beyamin, Rosenbaum (2021)	☺	?	☺	☹	☺	?	☺
Rajan et al (2023)	☺	☺	☺	?	☺	☺	☺

☺ Low Risk ☹ High Risk ? Unclear Risk

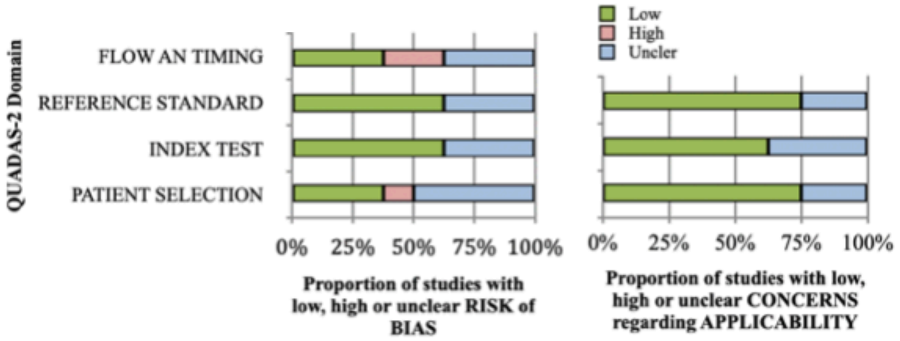

Supplement: Supplementary File 3. — QUADAS-2 Detailed Table. [file tohm-16-1-1151-s3.pdf]
